# Association of Communication Interventions to Discuss Code Status With Patient Decisions for Do-Not-Resuscitate Orders: A Systematic Review and Meta-analysis

**DOI:** 10.1001/jamanetworkopen.2019.5033

**Published:** 2019-06-07

**Authors:** Christoph Becker, Leopold Lecheler, Seraina Hochstrasser, Kerstin A. Metzger, Madlaina Widmer, Emanuel B. Thommen, Katharina Nienhaus, Hannah Ewald, Christoph A. Meier, Florian Rueter, Rainer Schaefert, Stefano Bassetti, Sabina Hunziker

**Affiliations:** 1 Department of Emergency Medicine, University Hospital Basel, Basel, Switzerland; 2 Medical Communication, Department of Psychosomatic Medicine, University Hospital Basel, Basel, Switzerland; 3 Clinic for Internal Medicine, University Hospital Basel, Basel, Switzerland; 4 Basel Institute for Clinical Epidemiology and Biostatistics, Department of Clinical Research, University Hospital Basel, Basel, Switzerland; 5 University Medical Library, University of Basel, Basel, Switzerland; 6 Quality Management, University Hospital Basel, Basel, Switzerland; 7 Medical Faculty, University of Basel, Basel, Switzerland

## Abstract

**Importance:**

Whether specific communication interventions to discuss code status alter patient decisions regarding do-not-resuscitate code status and knowledge about cardiopulmonary resuscitation (CPR) remains unclear.

**Objective:**

To conduct a systematic review and meta-analysis regarding the association of communication interventions with patient decisions and knowledge about CPR.

**Data Sources:**

PubMed, Embase, PsycINFO, and CINAHL were systematically searched from the inception of each database to November 19, 2018.

**Study Selection:**

Randomized clinical trials focusing on interventions to facilitate code status discussions. Two independent reviewers performed the data extraction and assessed risk of bias using the Cochrane Risk of Bias Tool. Data were pooled using a fixed-effects model, and risk ratios (RRs) with corresponding 95% CIs are reported.

**Data Extraction and Synthesis:**

The study was performed according to the PRISMA guidelines.

**Main Outcomes and Measures:**

The primary outcome was patient preference for CPR, and the key secondary outcome was patient knowledge regarding life-sustaining treatment.

**Results:**

Fifteen randomized clinical trials (2405 patients) were included in the qualitative synthesis, 11 trials (1463 patients) were included for the quantitative synthesis of the primary end point, and 5 trials (652 patients) were included for the secondary end point. Communication interventions were significantly associated with a lower preference for CPR (390 of 727 [53.6%] vs 284 of 736 [38.6%]; RR, 0.70; 95% CI, 0.63-0.78). In a preplanned subgroup analysis, studies using resuscitation videos as decision aids compared with other interventions showed a stronger decrease in preference for life-sustaining treatment (RR, 0.56; 95% CI, 0.48-0.64 vs 1.03; 95% CI, 0.87-1.22; between-group heterogeneity *P* < .001). Also, a significant association was found between communication interventions and better patient knowledge (standardized mean difference, 0.55; 95% CI, 0.39-0.71).

**Conclusions and Relevance:**

Communication interventions are associated with patient decisions regarding do-not-resuscitate code status and better patient knowledge and may thus improve code status discussions.

## Key Points

**Question:**

Is there an association between communication interventions and patient preference regarding do-not-resuscitate (DNR) code status decisions and knowledge regarding life-sustaining treatment?

**Findings:**

In this systematic review and meta-analysis, the pooled meta-analysis of 11 randomized clinical trials involving 1463 patients showed a significant association between communication interventions and higher patient preference for a DNR code status. In an analysis of 5 eligible trials, communication interventions were also associated with better patient knowledge about resuscitation.

**Meaning:**

Communication interventions may be an effective decision aid for code status discussions that potentially alter patient decisions regarding DNR code status and increase patient knowledge.

## Teaser

This systematic review and meta-analysis assesses the association of communication interventions about do-not-resuscitate code status with patient decisions and knowledge about cardiopulmonary resuscitation.

## Introduction

To inform patients about treatment options in case of a cardiac arrest and their involvement in the decision-making process regarding their code status is considered a cornerstone of patient-centered care.^1^ Physicians are encouraged to conduct such code status discussions to respect patient autonomy as an ethical principle.^2,3,4^ Also, it is important to ask hospitalized patients for their preference because cardiopulmonary arrest occurs in almost 1 per 1000 hospitalization days.^5^

However, the literature reports several shortcomings and challenges in conducting code status discussions. First, many patients have unrealistic expectations about cardiopulmonary resuscitation (CPR) and associated risks and benefits.^6,7^ Patients with in-hospital cardiac arrests generally have a poor prognosis, with a survival to hospital discharge rate less than 20%.^8,9^ Beyond, many survivors have substantial neurologic deficits, limiting the potential to live an independent life.^10^

However, physicians often omit code status discussions or do not describe resuscitation measures, such as chest compressions or mechanical ventilation.^11^ Although CPR is an invasive procedure with potential complications, risks and benefits are usually not communicated adequately to patients, contributing further to patient misconceptions.^12,13^

A recent study^14^ in patients with cancer found that physicians document a presumed code status rather than conduct a true discussion, leading to a high proportion of full code status. Almost one-third of patients who were documented as full code would have preferred a do-not-resuscitate (DNR) code status if adequately informed about the consequences of CPR.^14^

Moreover, code status discussions are often ineffective due to poor communication skills of physicians.^15,16^ This is particularly true for junior physicians, who conduct most of the code status discussions in clinical practice and often perceive themselves as unprepared to explain complex medical procedures.^17^ Furthermore, code status discussions are often conducted under time constraints in an impersonalized, procedure-focused way, missing the chance to focus on individual patient values and goals.^18,19,20,21^ A recent study^22^ from Switzerland found that treating physicians significantly altered patient choices, raising the question of patient autonomy.

To date, there is no consensus about the best approach to code status discussions to understand patient preference and choice regarding DNR code status. The objective of this systematic review and meta-analysis was to identify studies examining communication interventions designed to facilitate code status discussions. We were especially interested in the association of communication interventions with patient preference for CPR or DNR code status and knowledge regarding resuscitation and its outcome.

## Methods

### Types of Studies, Participants, and Outcome Measures

This systematic review and meta-analysis followed the Preferred Reporting Items for Systematic Reviews and Meta-analyses (PRISMA) reporting guidelines.^23^ We included randomized clinical trials (RCTs) in which the association of communication interventions during code status discussions with patient-relevant outcomes was compared with a control group. Studies were eligible if they focused on the outcomes of patient preference for resuscitation or DNR or patient knowledge regarding life-sustaining treatment.

### Search Terms for Identification of Studies

We performed a comprehensive search strategy consisting of a combination of Medical Subject Headings and free-text words. We searched PubMed, Embase, PsycINFO, and CINAHL.

We developed the search strategy in consultation with a medical librarian (H.E.) experienced in systematic reviews. Initial search terms were drawn from a small set of key articles. We used an iterative process of building a search strategy, running the search, scanning the relevant retrieved articles for additional terms, and then rebuilding the search strategy with the newly identified relevant terms and related Medical Subject Headings. Because we focused on RCTs, we also used a sensitivity and precision-maximizing RCT filter for our search.^24^ The final search strategy for PubMed, which was adapted for the other databases, is available in the Appendix (eTable 1 in the Supplement).

To identify additional published, unpublished, and ongoing studies, we (1) tracked relevant references through the cited reference search of Web of Science and PubMed, (2) applied the similar articles search of PubMed, and (3) screened all references of potentially eligible studies. The data search was performed between September 3 and November 19, 2018.

### Study Selection

Two of us (C.B. and L.L.) screened the titles and abstracts of articles found by the systematic search strategy. Studies were selected according to the inclusion criteria. We read the full texts of studies considered eligible for inclusion, and disagreement was resolved by discussion and consensus. Studies with the same assessment of end points were selected for quantitative meta-analysis regarding the association of communication interventions with primary and secondary end points.

### Data Extraction and Assessment of Methodological Quality

Two of us (C.B. and L.L.) independently extracted the data of the included studies. Relevant outcomes for our systematic review and meta-analysis were patient preference for resuscitation or DNR code status and knowledge regarding CPR.

The RCTs were assessed for methodological quality using the Cochrane Risk of Bias Tool to rate the risk of bias in random sequence generation, allocation concealment, selective reporting, masking, completeness of outcome data, and other possible bias^25^ (eTable 2 in the Supplement). If at least 1 of the domains was rated as high risk, the trial was considered at high risk of bias. If all domains were judged as low, the trial was considered to be at low risk of bias. Otherwise, the trial was considered at unclear risk of bias. Two of us (C.B. and L.L.) performed data extraction and risk of bias assessment independently; disagreement was resolved by involvement of a third author (S. Hunziker).

### Data Analysis

We express dichotomous data risk ratios (RRs) with 95% CIs and report continuous data as the mean differences with 95% CIs. Data were pooled using a fixed-effects model. We identified heterogeneity (inconsistency) through visual inspection of the forest plots. We used the *I*^2^ statistic, which quantifies inconsistency across studies, to assess the consequences of heterogeneity on the meta-analysis. An *I*^2^ statistic of 50% or more indicates a considerable level of heterogeneity. If data were not suitable for direct comparison, we applied narrative synthesis.

For the primary end point, we performed several predefined subgroup analyses that stratified the results based on the following: type of intervention (video intervention vs no video intervention), age (<75 vs ≥75 years), risk of bias according to the Cochrane Risk of Bias Tool, study setting (outpatients vs hospitalized patients), marital status (≤65% vs >65% of patients married), education of the population (>30% vs ≤30% with a college degree or higher), and sex (≤55% vs >55% male). These cutoffs for stratification were chosen post hoc based on the distribution among trials to achieve a balanced number of patients per group. For the secondary end point, we performed several predefined subgroup analyses stratifying the results based on age (<75 vs ≥75 years) and risk of bias.

Statistical analyses were performed using the METAN package in Stata (Stata MP, version 15.1; StataCorp LP). Two-sided *P* < .05 was considered statistically significant.

## Results

### Studies Identified

A total of 7001 records were identified through our database searches. We removed duplicates (n = 1203) and discarded 5206 studies after examining titles and 559 studies after screening abstracts. Of the remaining 33 full-text articles, 15 studies^26,27,28,29,30,31,32,33,34,35,36,37,38,39,40^ were eligible for inclusion (eFigure in the Supplement). Six studies were judged to be at low risk of bias, 4 studies at high risk of bias, and 5 studies at unclear risk of bias.

### Description of Studies

Table 1 lists characteristics of the 15 included RCTs. Publication dates ranged from 1999 to 2018, and studies were conducted mostly in the United States (14 trials^26,27,28,29,30,31,32,33,34,35,37,38,39,40^), with 1 trial^36^ from Australia. Across all studies, a total of 2405 participants were included, with study sample sizes ranging from 50 to 313 per trial. In 8 studies,^26,27,31,32,33,35,36,39^ participants were recruited among hospitalized patients, and a further 5 studies^28,29,34,38,40^ recruited outpatients, whereas 1 study^37^ investigated residents of a nursing facility and 1 study^30^ recruited outpatients and hospitalized patients.

**Table 1.** Summary of the Included Studies, With Quality Assessed Using the Cochrane Risk of Bias Tool

Source	Study Objective	Country	Participants	Design	Methods/Interventions		Detailed Communication/Intervention Elements	Primary End Point	Secondary End Points	Risk of Bias
					Intervention Group	Control Group				
Nicolasora et al,^33^ 2006	To detect whether hospitalized patients are willing to discuss end-of-life issues and choose whether to receive CPR and mechanical ventilation	United States	Patients on medical wards, hospitalized (N = 297)	RCT	Physicians approached patients with written scripts about CPR (n = 136)	Usual care (n = 161)	Script with information about life-sustaining therapy and advance directives. Patients were asked whether they wish to choose their CPR status	Usefulness of information in intervention group: 133/136 (98%) In intervention group willing to discuss CPR; 112/136 Found information useful; 6/136 Were disturbed; 3/136 Refused to discuss CPR	55/161 Patients in control group vs 125/136 in intervention group completed advance directives; 13/102 In intervention group vs 1/128 in control group (*P* < .001) completed advance directives	High
Stein et al,^36^ 2013	To determine whether an intervention could facilitate earlier DNR orders and reduce in-hospital deaths	Australia	Patients with metastatic cancer, without curative treatment and life expectancy 3-12 mo (N = 120)	RCT	Patients received a pamphlet and had a discussion with a psychologist (n = 55)	Usual care (n = 65)	Pamphlet “Living With Advanced Cancer” Discussion with psychologist was based on a shared decision-making model with the aim to encourage patients to consider their preferences and values toward the end of life	Place of death; Patients in intervention group less likely to die in hospital (7/36 vs 23/46; *P* < .01)	No. of DNR orders; 44 Patients (76%) in control group vs 26 patients (68%) in intervention group (*P* = .4); No. of days between DNR order and death; In intervention group, DNR orders were placed 2.2 (95% CI, 1.1-5.9; *P* = .03) times earlier; Hospital Anxiety and Depression Scale; No difference in anxiety or depression; The Caregivers Reaction Assessment; Knowledge questionnaire; Higher knowledge in intervention group after treatment and at follow-up	Low
El-Jawahri et al,^28^ 2010	To determine whether a video may facilitate end-of-life decision making for patients with cancer	United States	Patients with malignant glioma, outpatients (N = 50)	RCT	Patients had a discussion about end-of-life goals, after discussion patients were shown a video about the topic (n = 23)	Patients only had a discussion without the video (n = 27)	Baseline assessment of knowledge Discussion contained 3 levels of medical care in advanced cancer: life-prolonging care, basic medical care, and comfort care Video lasting 6 min	Preference for care; In control group, 7/27 (25.9%) preferred life-prolonging care vs 0/23 in intervention group (*P* < .001)	Preference for CPR; 11/27 In control group preferred CPR after intervention vs 2/23 in video group (*P* = .02); Patient Knowledge Score; Increase in knowledge in intervention group was 1.9 (95% CI, 1.3-2.4) vs 0.9 (95% CI, 0.4-1.3) in control group (*P* < .01); Level of uncertainty (Decisional Conflict Scale); 13.7 (95% CI, 12.8-14.6) in verbal group vs 11.5 (95% CI, 10.5-12.6) (*P* < .01)	Unclear
Volandes et al,^37^ 2012	To investigate the effect of a video decision support tool on CPR preferences of patients with advanced cancer	United States	Patients with advanced cancer, outpatients (N = 150)	RCT, multicenter	Viewed a video about resuscitation (n = 70)	Had normal discussion about CPR (n = 80)	Baseline questionnaire Verbal narrative describing CPR measures Was read to patient, language below an eighth grade reading level Video lasting 3 min showing real patient on ventilator Depiction of simulated CPR and intubation of a mannequin Immediate questionnaire after meeting; Knowledge score ranging from 0 to 4 Follow-up 6 and 8 wk by masked assistant	Preference for CPR; 38/80 In control group vs 14/70 in intervention group (*P* < .001)	Preference for CPR after 6 wk; 15/37 In control group vs 5/30 in intervention group (*P* = .06); Patient Knowledge Score; 2.6 ± 1.3 In control group vs 3.3 ± 1.0 (*P* < .001)	Low
El-Jawahri et al,^26^ 2015	To examine the effect of a video decision tool for CPR and intubation on patient choices, knowledge, medical orders, and discussions with health care providers	United States	Seriously ill patients, age >60 y, life expectancy <1 y, hospitalized patients (N = 150)	RCT	Video about CPR and intubation; assessment of participants’ preferences that were communicated to their treating physician (n = 75)	Usual care (n = 75)	Video lasting 3 min, depiction of simulated CPR and intubation of mannequin and patient receiving mechanical ventilation; Preferences of participants were communicated to treating medical team with note to confirm code status	Preference for CPR; 64% In control group vs 32% in intervention group (*P* < .001)	Participants’ preference for intubation; 72% In control group vs 43% in intervention group (*P* < .001); Patients with DNR orders at discharge; 57% In control group vs 19% in intervention group (*P* < .001); Code status discussions; 81% In intervention group vs 43% in control group (*P* < .001); Knowledge Score; 2.45 in control group vs 4.11 in intervention group (*P* < .001)	Low
Mittal et al,^32^ 2014	To determine the effect of a scripted code status explanation on patient understanding of choices	United States	Patients hospitalized to regular nursing floor, hospitalized patients (N = 300)	RCT	Discussion conductors provided standardized explanation on code status, advance directives, and end-of-life care (n = 150)	Usual care (n = 150)	Standardized code status explanation Based on review of the literature, approved by hospitalists Participating researchers received a briefing and simulation training Ad hoc questionnaire after discussion	Patient Knowledge Score; 5.27 In intervention group vs 4.93 in control group (*P* = .07)	NA	High
Rhondali et al,^34^ 2013	To determine the effect of a physician’s communication style, promoting patient autonomy vs promoting beneficence on patient preference regarding code status	United States	Patients with cancer who attended supportive care clinic, outpatients (N = 78)	RCT	First video, question at the end; patients saw a video regarding code status discussion that lasted 5 min (n = 35)	Second video, recommendation at the end; patients saw a video regarding code status discussion that lasted 5 min (n = 45)	First video ended with physician asking about code status preference Second video ended with physician recommending DNACPR Physicians played by actors; same actors in 2 videos	Preference for CPR; No difference in preference between 2 video types	No difference in perceived communication rating, physician impression score, or physician compassion score	Unclear
Merino et al,^31^ 2017	To determine the effect of a video about CPR on hospitalized patient code status choices	United States	Patients admitted to medical wards regardless of diagnoses/severity of illness, age >65 y, hospitalized patients (N = 119)	RCT	n = 59 Patients watched a 6-min video Participants circled preferred code status on a sheet Counseling by medical team if necessary	Usual care (n = 60)	Video explained different code choices (full code, DNR, DNR/DNI), demonstrating CPR on mannequin and palliative care specialists explaining potential complications and survival rates of resuscitation	Preference for CPR; 37% In intervention group and 71% in control group	Patients with DNR orders; 17% In control group vs 56% in intervention group (*P* < .0001); Trust in medical providers; 93% In control group vs 76% in intervention group had trust (*P* = .083)	Unclear
Wilson et al,^39^ 2015	To determine if a video showing CPR would improve knowledge and decision making among patients and surrogates	United States	ICU patients and their surrogates, within 48 h of admission, hospitalized patients (N = 208)	RCT	Video group plus usual care (n = 105)	Usual care (n = 103)	Usual care: 16-page pamphlet describing CPR measures and preference options Routine nonstandardized code status discussions with clinicians Video group: 8-min video; Production of a video on basis of clinicians and patients/relatives’ input Depiction of simulated CPR Information about complications and outcomes of CPR	Knowledge Score; 9 In intervention group vs 6.5 in control group (*P* < .0001)	Changes in resuscitation preferences; 5% In intervention group vs 6% in control group (*P* = 1); CPR within 30 d; 1% In intervention group vs 2% in control group (*P* = 1.00); Mechanical ventilation within 30 d; 17% In intervention group vs 13% in control group (*P* = .54)	Low
Richardson-Royer et al,^35^ 2018	To determine if a video augments script-only decision making regarding code status	United States	Patients admitted to medical wards, age >65 y, hospitalized patients (N = 100)	RCT	Video group plus standardized information (n = 105)	Standardized information (n = 103)	Standardized information Evidence-based information was read from a script to the patients Video showing a simulated CPR and mechanical ventilation	Preference for CPR; No difference between intervention group and control group (39/50 vs 39/50)	NA	Unclear
Epstein et al,^29^ 2013	To investigate whether a video with educational information about CPR leads to advance care planning	United States	Patients with pancreatic and hepatobiliary cancer, outpatients (N = 56)	RCT	Video about CPR (n = 30)	Narrative information about CPR (n = 26)	Video lasting 3 min: explanation of CPR/ventilation and its success rate in patients with advanced cancer Images of chest compressions, oxygenation via bag valve mask intubation, and ventilated patient on ICU Narrative arm: script identical to the one in the video	Advance directives after 30 d; 12 (40%) In intervention vs 4 (15%) in control group (*P* = .07)	Patient Knowledge Score; 4.9 In intervention group and 4.9 in control group (*P* = .746); Preference for CPR; 17% In intervention group vs 16% in control group (*P* = .61); Preference for ventilation; 21% In intervention group vs 12% in control group (*P* = .68)	Unclear
Volandes et al,^37^ 2012	To determine the effect of a video on preferences for the primary goal of care	United States	People living in nursing facilities after hospitalization, age >65 y (N = 101)	RCT	Video describing goals of care (n = 50)	Verbal narrative (n = 51)	Goals of care: Life prolonging: at any cost, including CPR Limited care: maintain physical functioning, intravenous fluids, antibiotic, no CPR Comfort care: maximize comfort and relieve pain Video lasting 6 min: showing images explaining the different goals of care (included ventilated patient on ICU, CPR on a mannequin) Verbal narrative: description of potential goals of care	Patient preference for comfort care; 80% In intervention group vs 57% in control group (*P* = .02)	Patient preference for CPR; 12% In intervention group vs 33% in control group	Low
El-Jawahri et al,^27^ 2016	To examine the effect of a video decision support tool and a patient checklist on advance care planning for patients with heart failure	United States	Participants with diagnosis of advanced heart failure and limited prognosis, age ≥64 y, hospitalized patients (N = 246)	RCT	n = 123 Verbal description Video description Checklist	Verbal description only (n = 123)	Video lasting 6 min describing goals of care in advanced heart failure. Checklist about advance care planning. Verbal description of goals of care in advanced heart failure	Life-prolonging care vs limited medical care vs comfort care; Intervention group, 22% life-prolonging, 25% limited, 63% comfort care, 2% uncertain vs 41% life-prolonging, 22% limited, 30% comfort care, 8% uncertain (*P* < .001)	Preference against CPR; Patients in intervention group more likely to forgo CPR (68% vs 35%; *P* < .001); Knowledge Score; 4.1 In intervention group vs 3.0 in control group (*P* < .001	Low
Yamada et al,^40^ 1999	To assess the effect of a multimedia, educational intervention about advance directives and CPR on the knowledge, attitude, and activity toward advance directives and CPR	United States	Veterans from outpatient clinic, age ≥70 y, outpatients (N = 117)	RCT	n = 62 Patients received handout about advance directives Patients received handout about CPR; patients watched a movie about advance directives	Patients received handout about advance directives (routine care) (n = 55)	Handouts Explaining CPR measures Outcome movie lasting 10 min Explained procedure of advance directives	Correct estimation of CPR survival; 62.9% In intervention group vs 32.0% in control group (*P* < .05)	Preference for CPR; 75.6% In intervention group vs 80.5% in control group; Completion of advance directives; 18.6% In intervention group vs 12.5% in control group	High
Kirchhoff et al,^30^ 2012	To assess the effect of advance care planning on identification of patient choices regarding care compared with usual care	United States	Patients with end-stage renal disease or congestive heart failure, outpatients and hospitalized patients (N = 313)	RCT	Patients received patient choices advance care planning (n = 160)	Patients received usual care; way of assessment of preference unclear (n = 153)	Intervention: Patients received interview by trained facilitator lasting 1.5 h to assist in advance care planning and ended in documentation of preferences Control: usual care included standard advance directives counseling	Preference for CPR; 74/160 (46%) In intervention group vs 59/153 (38.6%) in control group	Preferences for care; 37.7% In intervention group chose to withdraw from dialysis vs 17% in control group	High

Abbreviations: CPR, cardiopulmonary resuscitation; DNACPR, do not attempt cardiopulmonary resuscitation; DNI, do not intubate; DNR, do not resuscitate; ICU, intensive care unit; NA, not applicable; RCT, randomized clinical trial.

Eight studies^26,27,28,29,30,34,36,38^ used advanced diseases with a life expectancy less than 1 year, such as metastatic cancer, end-stage congestive heart, or renal failure, as the inclusion criteria, while 7 studies^31,32,33,35,37,39,40^ had no exclusion criteria based on illness. The mean age of the study population was 60 years or older in 12 studies. Six studies^26,27,31,35,37,40^ only recruited patients older than 60 or 65 years. Eleven studies assessed the outcome of preference for DNR of intervention vs control groups, and 8 studies assessed knowledge regarding CPR.

All studies used a dichotomous format (yes or no) to investigate the association of communication interventions with patient preference for CPR. Patient knowledge was assessed through questionnaires; 5 studies used the same questionnaire as in a previous study.^41^

Eleven included studies^26,27,28,29,31,34,35,37,38,39,40^ applied a video-based intervention. Ten videos showed simulated cardiac arrests and medical procedures undertaken during CPR, such as chest compressions and intubation. Some videos also contained images of real patients being treated on intensive care units, and other videos also provided information regarding end-of-life care or advance directives.^37,40^ Other studies used designed advance care planning interviews,^30^ standardized scripted explanations,^32,33^ or written information^36^ as interventions.

All studies used either structured questionnaires or interviews for data collection. One study^30^ did not specify assessment of preference for CPR.

### Quantitative Analysis

#### Primary End Point of Preference for CPR

**Figure 1.**
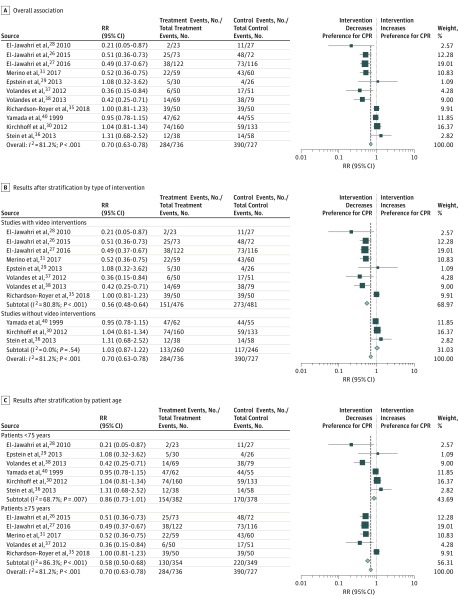
Forest Plot for the Association of Communication Interventions With Patient Preference for Resuscitation in 11 Trials^26,27,28,29,30,31,35,36,37,38,40^ The squares and horizontal lines correspond to the study-specific risk ratio (RR) and 95% CI. The diamond represents the pooled RR of overall preference. The vertical dashed line indicates the overall pooled RR of 0.70. CPR indicates cardiopulmonary resuscitation.

Of the 15 eligible trials, 4 did not report data regarding patient preference for resuscitation and were excluded from the quantitative analysis. The remaining 11 trials^26,27,28,29,30,31,35,36,37,38,40^ (1463 patients) were pooled for the meta-analysis (Figure 1).

Five of these 11 studies reported no significant association of interventions with patient preference for CPR, and 6 trials reported a significant decrease in preference for CPR. Compared with usual care, the pooled results showed a significant association between the communication interventions and a lower preference for CPR (390 of 727 [53.6%] vs 284 of 736 [38.6%]; RR, 0.70; 95% CI, 0.63-0.78). There was high heterogeneity among trials (*I*^2^ = 81.2%; *P* < .001).

#### Stratification by Type of Intervention, Age, Risk of Bias, Study Setting, Marital Status, Education, and Sex

To assess the association of communication interventions with patient preference for CPR in predefined subgroups, we stratified our results by type of intervention, age, risk of bias, study setting, marital status of participants, education, and sex (Table 2). When stratified by type of intervention, trials that used videos showing resuscitation as a decision aid in their intervention group compared with other types of interventions demonstrated a stronger decrease in preference for CPR (RR, 0.56; 95% CI, 0.48-0.64 vs 1.03; 95% CI, 0.87-1.22; between-group heterogeneity *P* < .001). Studies with low risk of bias had a stronger association with lower preference for CPR compared with trials with higher risk of bias (RR, 0.52; 95% CI, 0.43-0.63 vs 0.87; 95% CI, 0.76-0.99; between-group heterogeneity *P* < .001). Stratification by study setting also showed no difference between outpatients and hospitalized patients (RR, 0.64; 95% CI, 0.51-0.79 vs 0.71; 95% CI, 0.60-0.85; between-group heterogeneity *P* = .82). When stratified by marital status, the intervention had a stronger association with lower preference for CPR in trials with no more than 65% vs greater than 65% of patients being married (RR, 0.47; 95% CI, 0.38-0.58 vs 0.84; 95% CI, 0.50-1.39; between-group heterogeneity *P* = .02).

**Table 2.** Overall Results and the Results After Stratification of Meta-analysis

Variable	Preference for CPR				Knowledge			
	No. of Trials	Effect Size RR (95% CI)	Test for Heterogeneity		No. of Trials	Effect Size SMD (95% CI)	Test for Heterogeneity	
			*I*^2^ Statistic, %	*P* Value			*I*^2^ Statistic, %	*P* Value
Overall	11	0.70 (0.63 to 0.78)	81.2	<.001	5	0.55 (0.39 to 0.71)	53.9	.07
Stratified by type of intervention								
Video	8	0.56 (0.48 to 0.64)	80.8	<.001	NA	NA	NA	NA
No video	3	1.03 (0.87 to 1.22)	0.0	.54	NA	NA	NA	NA
Between-group heterogeneity	NA	NA	NA	<.001	NA	NA	NA	NA
Stratified by prognosis								
Poor	7	0.67 (0.57 to 0.78)	78.6	<.001	NA	NA	NA	NA
No poor known	4	0.77(0.66 to 0.89)	83.5	.003	NA	NA	NA	NA
Between-group heterogeneity	NA	NA	NA	<.001	NA	NA	NA	NA
Stratified by age, y								
<75	6	0.86 (0.73 to 1.01)	68.7	.007	3	0.48 (0.23 to 0.73)	51.6	.13
≥75	5	0.58 (0.50 to 0.68)	86.3	<.001	2	0.59 (0.39 to 0.80)	75.3	.04
Between-group heterogeneity	NA	NA	NA	.003				.48
Stratified by risk of bias								
High plus unclear	6	0.87 (0.76 to 0.99)	69.0	.007	2	0.28 (−0.10 to 0.67)	59.0	.12
Low	5	0.52 (0.43 to 0.63)	56.1	.06	3	0.60 (0.43 to 0.77)	50.8	.13
Between-group heterogeneity	NA	NA	NA	<.001				.14
Stratified by hospital setting								
Outpatients	5	0.64 (0.51 to 0.79)	83.1	<.001	NA	NA	NA	NA
Hospitalized patients	4	0.71 (0.60 to 0.85)	84.6	<.001	NA	NA	NA	NA
Between-group heterogeneity	NA	NA	NA	.82	NA	NA	NA	NA
Stratified by marital status, % of participants married								
>65	3	0.84 (0.50 to 1.39)	64.5	.06	NA	NA	NA	NA
≤65	4	0.47 (0.38 to 0.58)	0.0	.83	NA	NA	NA	NA
Between-group heterogeneity	NA	NA	NA	.02	NA	NA	NA	NA
Stratified by education, % of participants with college or university degree								
>30	4	0.94 (0.74 to 1.18)	47.8	.13	NA	NA	NA	NA
≤30	4	0.48 (0.39 to 0.59)	0.0	.83	NA	NA	NA	NA
Between-group heterogeneity	NA	NA	NA	<.001	NA	NA	NA	NA
Stratified by sex, % of participants male								
>55	4	0.49 (0.40 to 0.59)	0.0	.68	NA	NA	NA	NA
≤55	4	0.68 (0.54 to 0.85)	84.6	<.001	NA	NA	NA	NA
Between-group heterogeneity	NA	NA	NA	<.001	NA	NA	NA	NA

Abbreviations: CPR, cardiopulmonary resuscitation; NA, not applicable; RR, risk ratio; SMD, standardized mean difference.

Also, interventions had a stronger association with decreased preference for CPR in trials of patients with low education level (ie, ≤30% with college degree or higher) (RR, 0.48; 95% CI, 0.39-0.59 vs 0.94; 95% CI, 0.74-1.18; between-group heterogeneity *P* < .001). Regarding demographics, interventions had stronger association with reduced preference for CPR in trials that included older patients (≥75 years) compared with younger patients (RR, 0.58; 95% CI, 0.50-0.68 vs 0.86; 95% CI, 0.73-1.01; between-group heterogeneity *P* = .003) and in trials that had larger proportions of male patients (>55% vs ≤55% male) (RR, 0.49; 95% CI, 0.40-0.59 vs 0.68; 95% CI, 0.54-0.85; between-group heterogeneity *P* < .001).

### Secondary End Points

#### Key Secondary End Point of Knowledge

**Figure 2.**
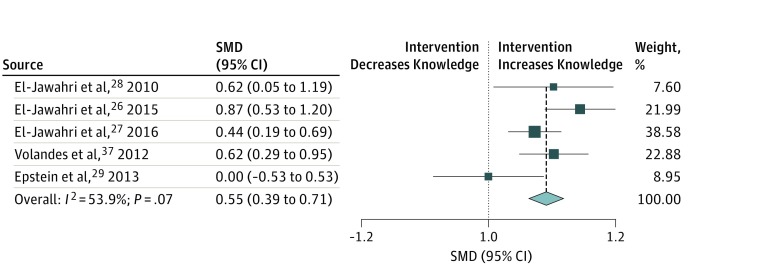
Forest Plot for the Association of Communication Interventions With Patient Knowledge Regarding Measures and Outcome of Resuscitation in 5 Trials^26,27,28,29,37^ The squares and horizontal lines correspond to the study-specific standardized mean difference (SMD) and 95% CI. The diamond represents the pooled SMD of patient knowledge. The vertical dashed line indicates the overall pooled SMD of 0.55.

Patient knowledge regarding CPR was assessed in 10 studies. Five trials used varying instruments to measure knowledge, which could not be standardized. We pooled the remaining 5 trials^26,27,28,29,37^ (including 652 patients) that used the exact same questionnaire for meta-analysis. In the pooled analysis, we found a significant association between communication interventions and higher patient knowledge (overall standardized mean difference [SMD], 0.55; 95% CI, 0.39-0.71). There was some heterogeneity among trials (*I*^2^ = 53.9%; *P* = .07) (Figure 2).

We then stratified the analysis by age and risk of bias. In low-risk trials, there was a stronger association between communication interventions and higher knowledge compared with higher-risk trials (SMD, 0.60; 95% CI, 0.43-0.77 vs 0.28; 95% CI, −0.10 to 0.67; between-group heterogeneity *P* = .14). Stratification by age did not show a significant difference between older and younger patients (SMD, 0.59; 95% CI, 0.39-0.80 vs 0.48; 95% CI, 0.23-0.73; between-group heterogeneity *P* = .48).

#### Other Patient-Relevant Outcomes

Three studies^29,33,40^ evaluated the associations of communication interventions with completion or presence of advance directives; however, they had too much heterogeneity to be included in a meta-analysis. Nicolasora et al^33^ assessed new completion rates of advance directives at hospital discharge and found that the intervention led to a significantly higher proportion of completed advance directives (0.8% vs 12.7%; *P* < .001). Yamada et al^40^ investigated the same topic 4 weeks after hospital discharge but also included patients who intended to fill out an advance directive, without reporting specific numbers. According to the authors, their results showed no significant findings. Epstein et al^29^ looked at advance care planning documentation overall, which included advance directives. It was not reported whether advance directives were completed in relation to the video intervention or whether they had already been in place before the study. The study found no statistical difference between the video intervention and control groups.

Several studies that used videos as decision aids assessed patient perception regarding the video intervention by ratings on a Likert-type scale.^26,27,28,29,37,39^ According to the results of those studies, patients generally were more comfortable watching a video, rating its content as useful or helpful in the process of decision making. One study^28^ used the Decisional Conflict Scale as a validated questionnaire to assess patient decision-making ability. In that study, the mean uncertainty score was significantly higher in the video group compared with the control group (13.7; 95% CI, 12.8-14.6 vs 11.5; 95% CI, 10.5-12.6; *P* = .002), indicating less uncertainty among patients who had seen the video in choosing between their treatment options.

#### Physician-Relevant Outcomes

One study^31^ investigating the effect of a video as a decision aid among 119 patients hospitalized on a general medical ward asked them about trust in their treating health care team as a secondary outcome. Trust was assessed on a 5-point Likert-type scale ranging from “agree” to “disagree.” There was no significant difference between groups (76% vs 93%; *P* = .08).

Rhondali et al^34^ investigated the extent to which patients perceived their physician as compassionate. Patients saw videos showing simulated code status discussions. Videos ended either with the physician making a recommendation or asking about patient preference. Independent of their allocated group, patients who opted for full code rated their physician as less compassionate than patients who opted for comfort care.

## Discussion

The findings of this systematic review and meta-analysis investigating associations between communication interventions to discuss code status and patient preference for resuscitation and patient knowledge regarding life-sustaining measures and outcome are 3-fold. First, we found a strong association between communication interventions and patient decisions regarding DNR code status, with lower preference for life-sustaining therapies if patients received a communication intervention compared with usual care. This association was more pronounced in studies with lower risk of bias. Second, associations between communication interventions and patient preference for a DNR code status were stronger when video-assisted decision aids were used, in trials that included older patients, in men, and among patients with lower healthy literacy. However, it is important to note that only a limited number of video interventions were tested in different settings. Third, communication interventions were also associated with better knowledge regarding resuscitation measures and the outcome of cardiac arrests. Again, trials with lower risk of bias had a stronger association with patient knowledge.

In line with our results demonstrating that more information delivered by communication interventions is associated with a higher probability for patients to choose a DNR code status, a previous trial^38^ found that health literacy (the ability to comprehend medical consequences) is a predictor of patient choice of DNR status. Therefore, more information may help patients make individualized informed decisions regarding resuscitation measures. Today, shared end-of-life decision making is considered an ethical obligation of patient-centered care to discuss equivalent treatment options, emphasizing patient autonomy and self-determination.^1,42,43,44^ However, decision making during code status discussions is often challenged by uncertainty surrounding interventions and therapies that might be available but whose outcomes remain uncertain.^45,46^ The results of the present systematic review and meta-analysis suggest that communication interventions, including video-assisted ones, enable patients to actively participate in the decision-making process by increasing their knowledge. This assumption is supported by a study^28^ using video that found a simultaneous increase in patient knowledge and decrease in decisional conflict regarding choice of care.

Previous studies^11,47,48^ reported variable quality of health care providers’ communication skills with hospitalized patients regarding code status. Herein, videos had the potential to inform patients in a standardized way and thereby promote shared decision making. However, interventions using visual components (eg, chest compressions, intubation, and ventilation) have been criticized because they may influence patients and lead them to a particular treatment choice. Furthermore, video tools as decision aids might not be applicable in some clinical settings due to limited accessibility and may be not suitable for elderly patients. Yet, some studies^26,27,28,29,37,38^ using Likert-type scales to assess patient comfort reported that patients were comfortable with watching a resuscitation video. However, there is also concern that videos as a decision aid might impair the patient-physician relationship. In 1 study,^31^ patients receiving a video intervention reported less trust in their treating health care team. In a study^4^ of advance care planning interventions, patients who opted for life-sustaining treatment perceived their physician as less compassionate, suggesting that these patients might not have approved of the video approach. Hence, video-assisted interventions may be useful adjuvants for code status discussions but should not be a substitute for direct patient-physician communication. A more flexible approach that can be adapted to individual patient needs might be more favorable and easier to implement in busy clinical environments.

A 2012 British multicenter cohort study^49^ investigated medical records of patients who had undergone resuscitation after an in-hospital cardiac arrest. In more than 75% of patients who received CPR, the code status was unknown, and 67% of patients who were resuscitated had an underlying preexisting fatal disease. An independent post hoc assessment of all cases found that a DNR status would have been appropriate in 85% because the risk-benefit ratio was unfavorable for these patients. As in patients with diseases for which they are receiving palliative care, CPR is not beneficial and may even prolong the dying process.^3^ In addition, we found that the interventions of our studies herein were associated with a greater reduction in patient preference for CPR in patients 75 years or older compared with younger patients. Also, in patients with a poor prognosis, we observed that the interventions had a stronger association with patient choice of a DNR code status. Therefore, such patients may receive the most benefit from communication interventions.

In general, a patient decision regarding DNR code status is a legal order to withhold CPR or advanced cardiac life support in case of cardiac arrest or respiratory failure and has important medical and socioeconomic consequences.^50,51^ Those 2 systematic reviews found variability in DNR decision making and implementation of DNR code status, leading to suboptimal care with undesired withdrawal of treatment in case of clinical deterioration. A standardized decision-making and documentation process of code status discussions may thus help improve quality of care and enable physicians to make decisions in the best interest of their patients. Today, an increasing number of hospitals and care centers use medical decision systems, such as Physician Orders for Scope of Treatment (POLST), Medical Orders for Scope of Treatment (MOLST), or Recommended Summary Plan for Emergency Care and Treatment (ReSPECT), which embed treatment plans in case of clinical deterioration. Based on our findings, it would be relevant to integrate communication interventions into such decision systems to further improve the uniformity of clinical care and strengthen patient involvement in the decision process.

In 1995, the Study to Understand Prognoses and Preferences for Outcomes and Risks of Treatments (SUPPORT),^52^ a landmark trial to investigate different approaches to improve care for seriously ill patients, reported shortcomings in communication during code status discussions. Despite all research efforts over more than 20 years, there is still need for large and high-quality RCTs focusing on interventions to facilitate code status discussions. In our systematic review and meta-analysis, we found only 3 studies^30,32,33^ that investigated interventions other than videos on patient preference for care and knowledge regarding resuscitation. There is clearly need for further trials regarding this important topic.

### Limitations

We are aware of several limitations to this systematic review and meta-analysis. The meta-analysis is based on a small number of trials and patients that could be considered for the quantitative analysis, and additional research is needed to confirm these results. A large proportion of trials targeted a population of terminally ill patients with a life expectancy less than 1 year, and generalizability to other patient populations is thus limited. In addition, the study populations were similar regarding ethnicity (mostly white) and age group (most were aged ≥60 years), again limiting generalizability of our results. Furthermore, most trials were performed in the United States, limiting transferability to other populations due to differences in medical and socioeconomic systems. Also, 5 of our 15 RCTs were performed by the same 2 groups of investigators (ie, by El-Jawahri et al^26,27,28^ and by Volandes et al^37,38^), and the findings from their trials had stronger effects compared with trials from other groups regarding patient preference for a DNR code status. However, those 5 studies had low risk of bias, and trials were performed in different settings (ie, outpatients vs hospitalized patients) and with different patient populations (ie, those with palliative vs curative diseases). Therefore, validation of our results by independent research groups is warranted. The number of trials and patients was small, also limiting interpretation of our subgroup analyses and increasing the risk for type II error.

## Conclusions

Communication interventions may be an effective decision aid for code status discussions, potentially altering patient preference and increasing patient knowledge. More informed patients may be better able to participate in the decision-making process, which might prevent unwanted excessive medical procedures. There is still urgent need for large-scale RCTs to investigate further approaches to facilitate code status discussions.

## Notes

Supplement. **eTable 1.** Search Strategy for PubMed **eTable 2.** Risk Assessment by Cochrane Risk of Bias Tool **eReferences.** **eFigure.** Flow of Studies Through the Review Process
