# Supplementary material for: Association of Communication Interventions to Discuss Code Status With Patient Decisions for Do-Not-Resuscitate Orders: A Systematic Review and Meta-analysis
Source: JAMA Netw Open. 2019 Jun 7;2(6):e195033. doi: 10.1001/jamanetworkopen.2019.5033 (PMC6563579; doi:10.1001/jamanetworkopen.2019.5033)
Supplement: Supplement. — eTable 1. Search Strategy for PubMed eTable 2. Risk Assessment by Cochrane Risk of Bias Tool eReferences. eFigure. Flow of Studies Through the Review Process [file jamanetwopen-2-e195033-s001.pdf]

Supplementary Online Content

Becker C, Lecheler L, Hochstrasser S, et al. Association of communication interventions to discuss code status with patient decisions for do-not-resuscitate orders: a systematic review and meta-analysis. *JAMA Netw Open*. 2019;2(6):e195033. doi:10.1001/jamanetworkopen.2019.5033

eTable 1. Search Strategy for PubMed

eTable 2. Risk Assessment by Cochrane Risk of Bias Tool

eReferences.

eFigure. Flow of Studies Through the Review Process

This supplementary material has been provided by the authors to give readers additional information about their work.

© 2019 Becker C et al. *JAMA Network Open*.

eTable 1. Search Strategy for PubMed
Search date from inception to 19 November 2018; Hits: 2805

(resuscitation[mh] OR resuscitat*[tiab] OR do-not-resuscitate[tiab] OR CPR[tiab] OR Code Blue[tiab] OR code status[tiab] OR (Life[tiab] AND Prolong*[tiab]) OR cardiopulmonary arrest[tiab] OR heart massage[tiab] OR cardiac massage[tiab])
AND
(decision making[tiab] OR Decision Making[mh] OR decision aid[tiab] OR decision support[tiab] OR "Decision Support Systems, Clinical"[mh] OR decision support[tiab] OR communicat*[tiab] OR conversation[tiab] OR dicuss*[tiab] OR dialogue[tiab] OR educat*[tiab] OR "Patient Education Handout" [Publication Type] OR "Patient Education as Topic"[Mesh:noexp] OR "Health Communication"[mh] OR intervention[tiab] OR interventions[tiab])
AND
(randomized controlled trial[pt] OR controlled clinical trial[pt] OR randomized[tiab] OR placebo[tiab] OR "clinical trials as topic"[MeSH Terms:noexp] OR randomly[tiab] OR trial[ti] NOT ("animals"[MeSH Terms] NOT "humans"[MeSH Terms]))

Search results for other Databases

EMBASE via EMBASE (search date from inception to 19 November 2018); Hits: 3206

PsycINFO via PsycINFO (search date from inception to 19 November 2018); Hits: 835

CINAHL via CINAHL (search date from inception to 19 November 2018); Hits; 155

eTable 2. Risk Assessment by Cochrane Risk of Bias Tool

Green colour denotes low risk of bias, red colour denotes high risk of bias, yellow colour denotes unclear risk of bias.

AUTHOR / YEAR	STUDY NAME	RANDOM SEQUENCE GENERATION	ALLOCATION CONCEALMENT	SELECTIVE REPORTING	OTHER BIAS	BLINDING OF PARTICIPANTS AND PERSONNEL	BLINDING OF OUTCOME ASSESSMENT	INCOMPLETE OUTCOME DATA	RISK OF BIAS
**Nicolasora, Nelson et al 2006[1]**	**If Asked, Hospitalized Patients Will Choose Whether to Receive Life-Sustaining Therapies**	Usage of random number generator	Usage/method of concealment not described	Insufficient information to permit judgement	Control group was not approached by study team	Outcome is not likely to be influenced by lack of blinding	Outcome measurement is not likely to be influenced by lack of blinding	No missing outcome data	HIGH
**Stein, Rhea A. et al 2013[2]**	**Randomized Controlled Trial of a Structured Intervention to Facilitate End-of-Life Decision Making in Patients With Adcanced Cancer**	Usage of computer randomization scheme	Randomization was concealed	Insufficient information to permit judgement	Study seems to be free of other sources of bias	Outcome is not likely to be influenced by lack of blinding	Outcome measurement is not likely to be influenced by lack of blinding	Reasons for missing outcome data unlikely to be related to true outcome / Missing outcome data balanced across groups / Missing data appropiately imputed	LOW
**El-Jawahri, Areej et al 2010 [3]**	**Use of Video to Facilitate End-of-Life Discussions With Patients With Cancer: A Randomized Controlled Trial**	Usage of computer randomization scheme	Usage of envelopes for concealment described but unkown if sequentially numbered, opaque and sealed	Insufficient information to permit judgement	Study seems to be free of other sources of bias	Outcome is not likely to be influenced by lack of blinding	Outcome measurement is not likely to be influenced by lack of blinding	No missing outcome data	UNCLEAR
**Volandes, Angelo E. et al 2012[4]**	**Randomized Controlled Trial of a Video Decision Support Tool for Cardiopulmonary**	Usage of computer randomization scheme	Usage of envelopes for concealment described but unkown if	Study protocol available , pre-specified outcomes	Study seems to be free of other	Outcome is not likely to be influenced by lack of blinding	Outcome measurement is not likely to be	Reasons for missing outcome data unlikely to be related to true	LOW

© 2019 Becker C et al. *JAMA Network Open*.

	**Resuscitation Decision Making in Advanced Cancer**		sequentially numbered, opaque and sealed	reported (NCT01241929)	sources of bias		influenced by lack of blinding	outcome / Missing outcome data balanced across groups	
**El-Jawahri, Areej et al 2015[5]**	**A Randomized Controlled Trial of a CPR and Intubation Video Decision Support Tool for Hospitalized Patients**	Usage of computer randomization scheme	Randomization was concealed with numbered envelopes	Study protocol available , pre-specified primary and secondary outcomes relevant to review reported (NCT01325519)	Study seems to be free of other sources of bias	Outcome is not likely to be influenced by lack of blinding	Outcome measurement is not likely to be influenced by lack of blinding	No missing outcome data (Prim.EP), no information if missing data of sec. EP but unlikely to be related to true outcome	LOW
**Mittal, Kriti et al 2014[6]**	**Use of a standardized code status explanation by residents among hospitalized patients**	Insufficient information about the sequence generation process to permit judgemen	Usage/method of concealment not described	Insufficient information to permit judgement	Study seems to be free of other sources of bias	Outcome is not likely to be influenced by lack of blinding	Outcome measurement is not likely to be influenced by lack of blinding	Reasons for missing outcome data unlikely to be related to true outcome / Missing outcome data balanced across groups	HIGH
**Rhondali, Wadih et al 2013[7]**	**Patient-Physician Communication About Code Status Preferences**	Usage of computer randomization scheme	Method of concealment not described	Insufficient information to permit judgement	Study seems to be free of other sources of bias	Outcome is not likely to be influenced by lack of blinding	Outcome measurement is not likely to be influenced by lack of blinding	Reasons for missing outcome data unlikely to be related to true outcome	UNCLEAR
**Merino, Aimee M. et al 2017[8]**	**A Randomized Controlled Trial of a CPR Decision Support Video for Patients Admitted to the General Medicine Service**	Usage of random number generator	Usage/method of concealment not described	Insufficient information to permit judgement	Study seems to be free of other sources of bias	Outcome is not likely to be influenced by lack of blinding	Outcome measurement is not likely to be influenced by lack of blinding	Reasons for missing outcome data unlikely to be related to true outcome	UNCLEAR
**Wilson, ME et al 2015[9]**	**A video to improve patient and surrogate understanding of cardiopulmonary resuscitation**	Usage of random number generator	Randomization was concealed before assessment with sequentially numbered	Study protocol available , pre-specified primary outcomes reported	Study seems to be free of other sources of bias	Outcome is not likely to be influenced by lack of blinding	Outcome measurement is not likely to be influenced by lack of blinding	Reasons for missing outcome data unlikely to be related to true outcome	LOW

© 2019 Becker C et al. *JAMA Network Open*.

	**choices in the ICU: a randomized controlled trial**		opaque sealed envelopes	(NCT10-003628)				/ Missing outcome data balanced across groups	
**Richardson-Royer, C et al 2018[10]**	**A video depicting resuscitation did not impact upon patients' decision making**	Method of randomization not described	Usage/method of concealment not described	Study protocol available, relevant pre-specified primary outcomes reported (NCT01878968)	Study seems to be free of other sources of bias	Outcome is not likely to be influenced by lack of blinding	Outcome measurement is not likely to be influenced by lack of blinding	No missing outcome data	UNCLEAR
**Epstein, Andrew S 2013[11]**	**A Randomized Controlled Trial oif a Cardiopulmonary Resuscitation Video in Advance Care Planning for Progressive Pancreas and Hepatobiliary Cancer Patients**	Usage of permuted block randomization	Usage/method of concealment not described	Insufficient information to permit judgement	Study seems to be free of other sources of bias	Outcome is not likely to be influenced by lack of blinding	Outcome measurement is not likely to be influenced by lack of blinding	Reasons for missing outcome data unlikely to be related to true outcome / Missing outcome data balanced across groups	UNCLEAR
**Volandes, Angelo E. 2012[12]**	**A Randomized Controlled Trial of a Goals-of-Care Video for Elderly Patients Admitted to Skilled Nursing Facilities**	Usage of computer randomization scheme	Usage of envelopes for concealment described but unkown if sequentially numbered, opaque and sealed	Study protocol available, relevant pre-specified primary outcome reported (NCT01233973)	Study seems to be free of other sources of bias	Outcome is not likely to be influenced by lack of blinding	Outcome measurement is not likely to be influenced by lack of blinding	Reasons for missing outcome data unlikely to be related to true outcome / Missing outcome data balanced across groups	LOW
**El-Jawahri, Areej et al 2016[13]**	**A Randomized Controlled Trial of an Advance Care Planning Video Decision Support Tool for Patients with Advanced Heart Failure**	Usage of computer randomization scheme	Usage of envelopes for concealment described but unkown if sequentially numbered, opaque and sealed	Study protocol available , pre-specified outcomes reported (NCT01589120)	Study seems to be free of other sources of bias	Outcome is not likely to be influenced by lack of blinding	Outcome measurement is not likely to be influenced by lack of blinding	Reasons for missing outcome data unlikely to be related to true outcome / Missing outcome data balanced across groups	LOW

© 2019 Becker C et al. *JAMA Network Open*.

**Yamada, Ryo et al 1999**[14]	**A Multemedia Intervention on Cardiopulmonary Resuscitation and Advance Directives**	Randomization based on clinic half-day and the week of the month	Usage/method of concealment not described	Insufficient information to permit judgement	Study seems to be free of other sources of bias	Outcome is not likely to be influenced by lack of blinding	Outcome measurement is not likely to be influenced by lack of blinding	Insufficient reporting of missing data	HIGH
**Kirchhoff et al 2012**[15]	**Impact of a Disease-Specific Advance Care PlanningIntervention on End-of-life Care**	Method of randomization not described	Usage of sealed-envelope method for concealment described but unkown if sequentially numbered and opaque	Insufficient information to permit judgement	Surrogates involved in intervention; Relevant outcome only for deceased patients way of assessment of outcome unclear	Outcome is not likely to be influenced by lack of blinding	Outcome measurement is not likely to be influenced by lack of blinding	No missing outcome data	HIGH

© 2019 Becker C et al. *JAMA Network Open*.

References

1. Nicolasora N, Pannala R, Mountantonakis S, et al. If asked, hospitalized patients will choose whether to receive life-sustaining therapies. *J Hosp Med.* 2006;1(3):161-167.
2. Stein RA, Sharpe L, Bell ML, Boyle FM, Dunn SM, Clarke SJ. Randomized controlled trial of a structured intervention to facilitate end-of-life decision making in patients with advanced cancer. *J Clin Oncol.* 2013;31(27):3403-3410.
3. El-Jawahri A, Podgurski LM, Eichler AF, et al. Use of video to facilitate end-of-life discussions with patients with cancer: a randomized controlled trial. *J Clin Oncol.* 2010;28(2):305-310.
4. Volandes AE, Paasche-Orlow MK, Mitchell SL, et al. Randomized controlled trial of a video decision support tool for cardiopulmonary resuscitation decision making in advanced cancer. *J Clin Oncol.* 2013;31(3):380-386.
5. El-Jawahri A, Mitchell SL, Paasche-Orlow MK, et al. A Randomized Controlled Trial of a CPR and Intubation Video Decision Support Tool for Hospitalized Patients. *J Gen Intern Med.* 2015;30(8):1071-1080.
6. Mittal K, Sharma K, Dangayach N, et al. Use of a standardized code status explanation by residents among hospitalized patients. *J Community Hosp Intern Med Perspect.* 2014;4.
7. Rhondali W, Perez-Cruz P, Hui D, et al. Patient-physician communication about code status preferences: a randomized controlled trial. *Cancer.* 2013;119(11):2067-2073.
8. Merino AM, Greiner R, Hartwig K. A Randomized Controlled Trial of a CPR Decision Support Video for Patients Admitted to the General Medicine Service. *J Hosp Med.* 2017;12(9):700-704.
9. Wilson ME, Krupa A, Hinds RF, et al. A video to improve patient and surrogate understanding of cardiopulmonary resuscitation choices in the ICU: a randomized controlled trial. *Crit Care Med.* 2015;43(3):621-629.
10. Richardson-Royer C, Naqvi I, Riffel C, et al. A video depicting resuscitation did not impact upon patients' decision-making. *Int J Gen Med.* 2018;11:73-77.
11. Epstein AS, Volandes AE, Chen LY, et al. A randomized controlled trial of a cardiopulmonary resuscitation video in advance care planning for progressive pancreas and hepatobiliary cancer patients. *J Palliat Med.* 2013;16(6):623-631.
12. Volandes AE, Brandeis GH, Davis AD, et al. A randomized controlled trial of a goals-of-care video for elderly patients admitted to skilled nursing facilities. *J Palliat Med.* 2012;15(7):805-811.
13. El-Jawahri A, Paasche-Orlow MK, Matlock D, et al. Randomized, Controlled Trial of an Advance Care Planning Video Decision Support Tool for Patients With Advanced Heart Failure. *Circulation.* 2016;134(1):52-60.
14. Yamada R, Galecki AT, Goold SD, Hogikyan RV. A multimedia intervention on cardiopulmonary resuscitation and advance directives. *J Gen Intern Med.* 1999;14(9):559-563.
15. Kirchhoff KT, Hammes BJ, Kehl KA, Briggs LA, Brown RL. Effect of a disease-specific advance care planning intervention on end-of-life care. *J Am Geriatr Soc.* 2012;60(5):946-950.

© 2019 Becker C et al. *JAMA Network Open*.

eFigure. Flow of Studies Through the Review Process

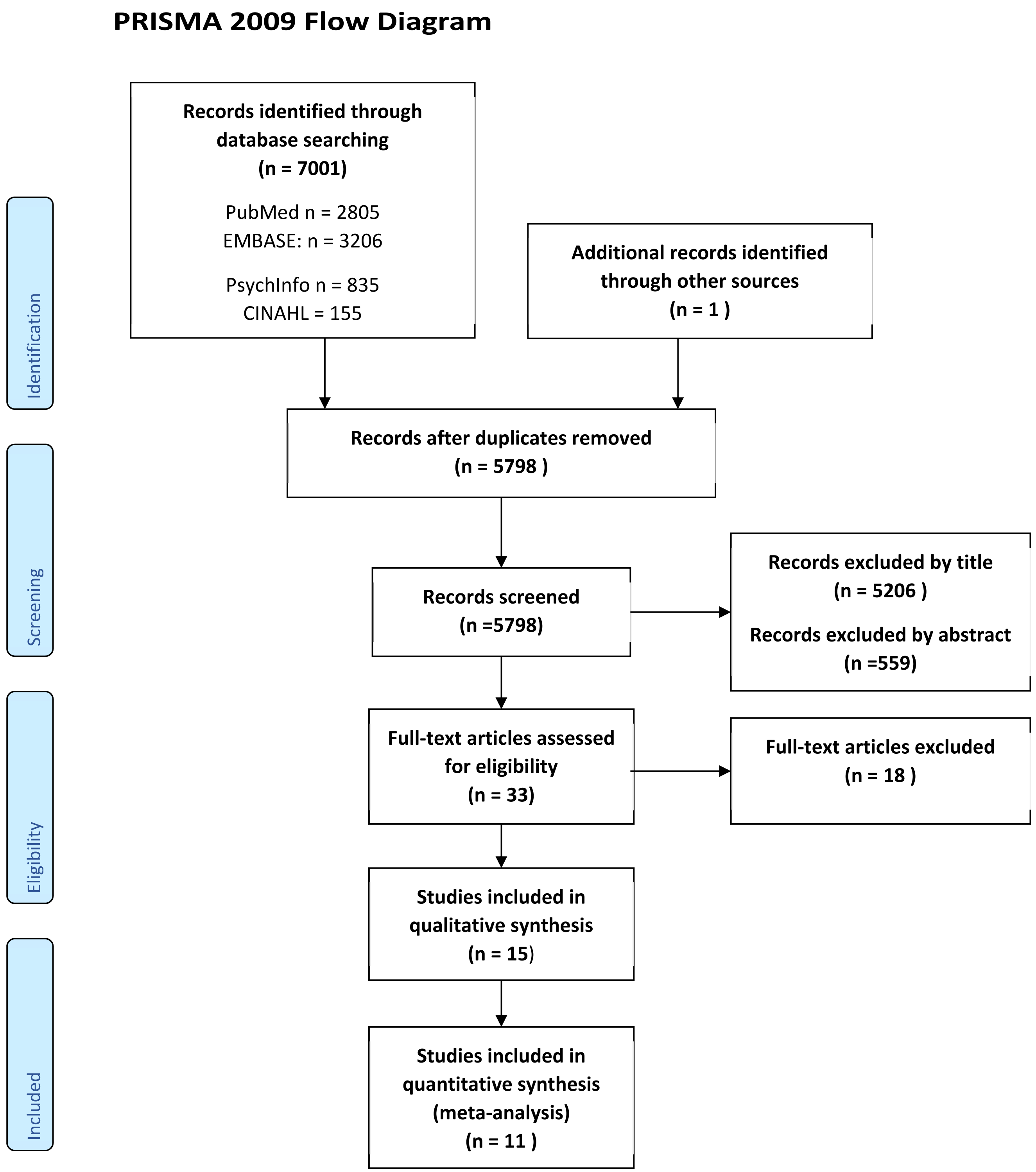

© 2019 Becker C et al. *JAMA Network Open*.
